# Case Report: Pulmonary alveolar adenoma: a case series from a single institution and literature review

**DOI:** 10.3389/fonc.2026.1887451

**Published:** 2026-06-29

**Authors:** Martina Maione, Jose Coelho Lima, Giuseppe Maggioni, Chiara Giraudo, Federica Pezzuto, Fiorella Calabrese

**Affiliations:** 1 Department of Medicine (DIMED), University of Padua, Padua, Italy; 2 Histopathology Department, Cambridge University Hospital, National Health Service (NHS) Foundation Trust, Cambridge, United Kingdom; 3 PhD Program in Translation Specialistic Medicine “G.B. Morgagni”, Curriculum “Thoracic and Pulmonary Sciences”, University of Padua, Padua, Italy; 4 Department of Cardiac, Thoracic, Vascular Sciences and Public, University of Padua, Padua, Italy

**Keywords:** alveolar adenoma, benign pulmonary neoplasm, immunohistochemistry, molecular profiling, next-generation sequencing

## Abstract

Alveolar adenomas are extremely rare benign epithelial lung neoplasms. They usually present as incidental radiologic findings in asymptomatic or paucisymptomatic patients. We report two cases observed at our institution over a two-year period.

## Introduction

Alveolar adenoma is an extremely rare pathological entity, first described in 1986 by Yousem et al. (1). Since then, to our knowledge, approximately seventy cases have been reported in literature. The exact incidence of these tumors is challenging to determine due to the paucity of case reports. It is estimated that they account for less than 1% of all pulmonary neoplasms. Alveolar adenoma is considered a benign lesion and is most often identified as an incidental finding in asymptomatic patients, more often middle-aged women. In some cases, however, it may be associated with respiratory symptoms such as chest pain, dyspnea, and cough. Reports of co-existence with other synchronous neoplasms are extremely rare in literature. Here, we present two challenging cases of alveolar adenoma, one that clinically and radiologically mimicked a metastasis from a synchronous breast carcinoma and the other as an incidental finding in a young patient with end-stage chronic kidney disease, awaiting for a transplantation, highlighting the importance of this entity in the differential diagnosis with both primary lung neoplasm and metastasis to the lung. To date, comprehensive molecular profiling using next-generation sequencing (NGS) has not been performed in these neoplasms. We describe the tumors’ histologic, immunohistochemical, and molecular profiles and review the literature.

## Case description

Case 1. A 55-year-old female with left breast carcinoma underwent a chest Computed Tomography (CT) for radiologic staging of the neoplasm, which evidenced a 13 mm hypodense solitary nodule in the left lower lobe (LLL) base consistent with a metastasis from the primary breast cancer. There were no other pulmonary nodules or mediastinal and/or axillary lymphadenopathy. The patient was a non-smoker and had a previous medical history that included obesity, systemic arterial hypertension, a benign thyroid nodule, and fibromyalgia. The patient underwent 6 cycles of neo-adjuvant chemotherapy followed by left breast quadrantectomy and axillary lymph node clearance. After the surgical treatment, adjuvant chemoradiotherapy and 3-monthly radiologic surveillance of the solitary lung nodule were performed. A positron emission tomography (PET) scan performed at approximately one year post-surgery showed no significant avidity for 18-fluorodeoxyglucose (18-FDG) by the lung nodule or lymph nodes. However, a subsequent chest CT performed 6 months later evidenced an increase (5 mm) in size of the lung nodule to 18 mm. There was no mediastinal or axillary lymphadenopathy. Following multidisciplinary team discussion, a decision was made to proceed with surgical resection of the lung nodule with left lower lobe segmentectomy and mediastinal lymphadenectomy due to the increase in size of the lesion.

**Figure 1:**
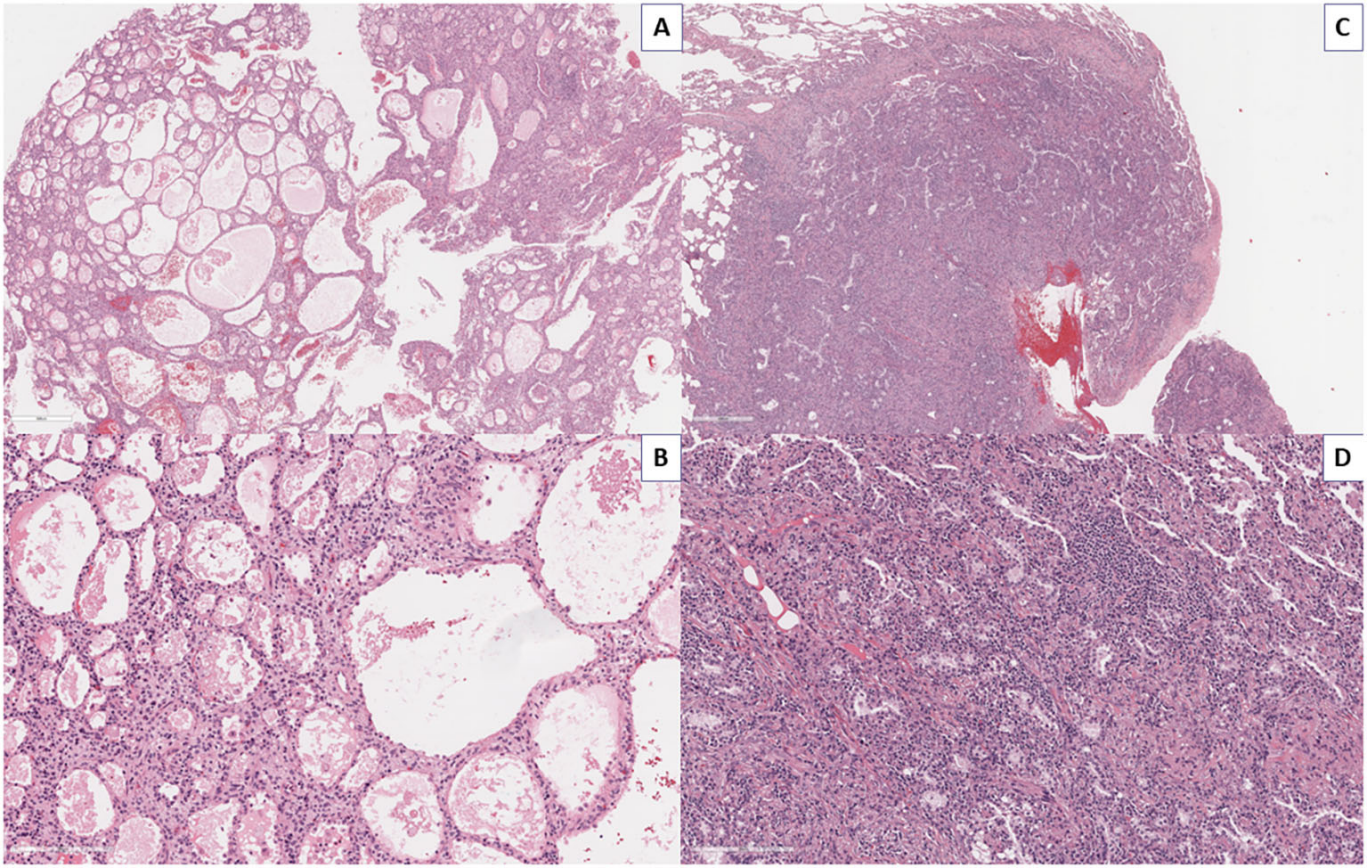
Histologic examination of the surgical specimens revealed, in both cases, a well circumscribed proliferation in the lung parenchyma [**(A)**, hematoxylin and eosin of case 1, scale bar: 600 μm; **(C)**, hematoxylin and eosin of case 2, scale bar: 600 μm]. At higher magnification, the tumors were composed of cystic spaces lined by epithelial cells and a stromal component composed of spindle cells. Case 1 shows a proliferation characterized by well-defined cystic and microcystic spaces embedded within a moderately cellular stroma composed of ovoid cells. In contrast, Case 2 shows a proliferation in which the microcystic spaces are predominantly collapsed, while the stroma consists of paucicellular fibrous bands containing spindle cells. [**(B)**, hematoxylin and eosin of case 1, scale bar: 200 μm; **(D)**, hematoxylin and eosin of case 2, scale bar: 200 μm].

On gross examination, the nodule was well circumscribed, partly cystic and solid and bulged from the surrounding lung parenchyma. Histologically, the tumor was non-encapsulated but well-demarcated from the adjacent lung tissue. The neoplasm was composed of variably-sized cystic spaces interspersed by solid cellular areas. The cystic spaces contained abundant eosinophilic granular material and were lined by a single layer of cuboidal to hobnailed cells resembling type II pneumocytes. Some of the cystic spaces contained abundant red blood cells or foamy macrophage clusters. Ciliated cells or a double cell layer were not a feature. The intervening solid areas comprised spindle cells admixed with lymphocytes, plasma cells, and conspicuous eosinophils within a delicate connective tissue stroma. No significant nuclear pleomorphism, conspicuous mitotic activity, necrosis, heterologous elements (i.e. cartilage, adipose tissue, smooth muscle), invasive growth or lymph node metastases were present (**Figure 1**).

**Figure 2:**
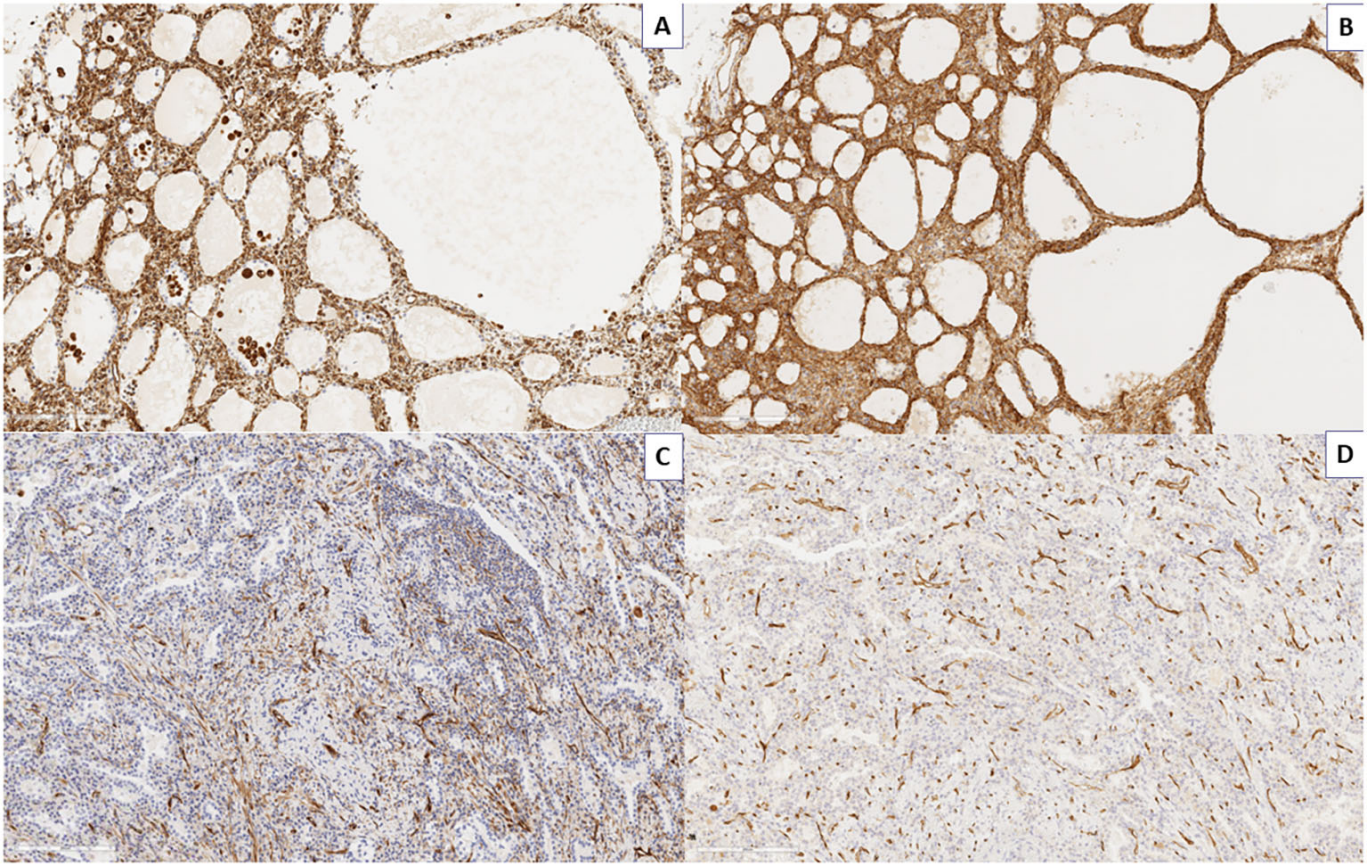
Immunohistochemistry revealed, in both cases, Vimentin and CD34 positivity of the stromal component. While case 1 shows a strong and diffuse positivity for both the immunoistochemical markers, case 2 exhibit only a focal and weak positivity for Vimentin and CD34 [**(A)** case 1, Vimentin, scale bar: 200 μm; **(B)** case 1, CD34, scale bar: 200 μm; **(C)** case 2, Vimentin, scale bar: 200 μm; **(D)** case 2, CD34, scale bar: 200 μm].

**Figure 3:**
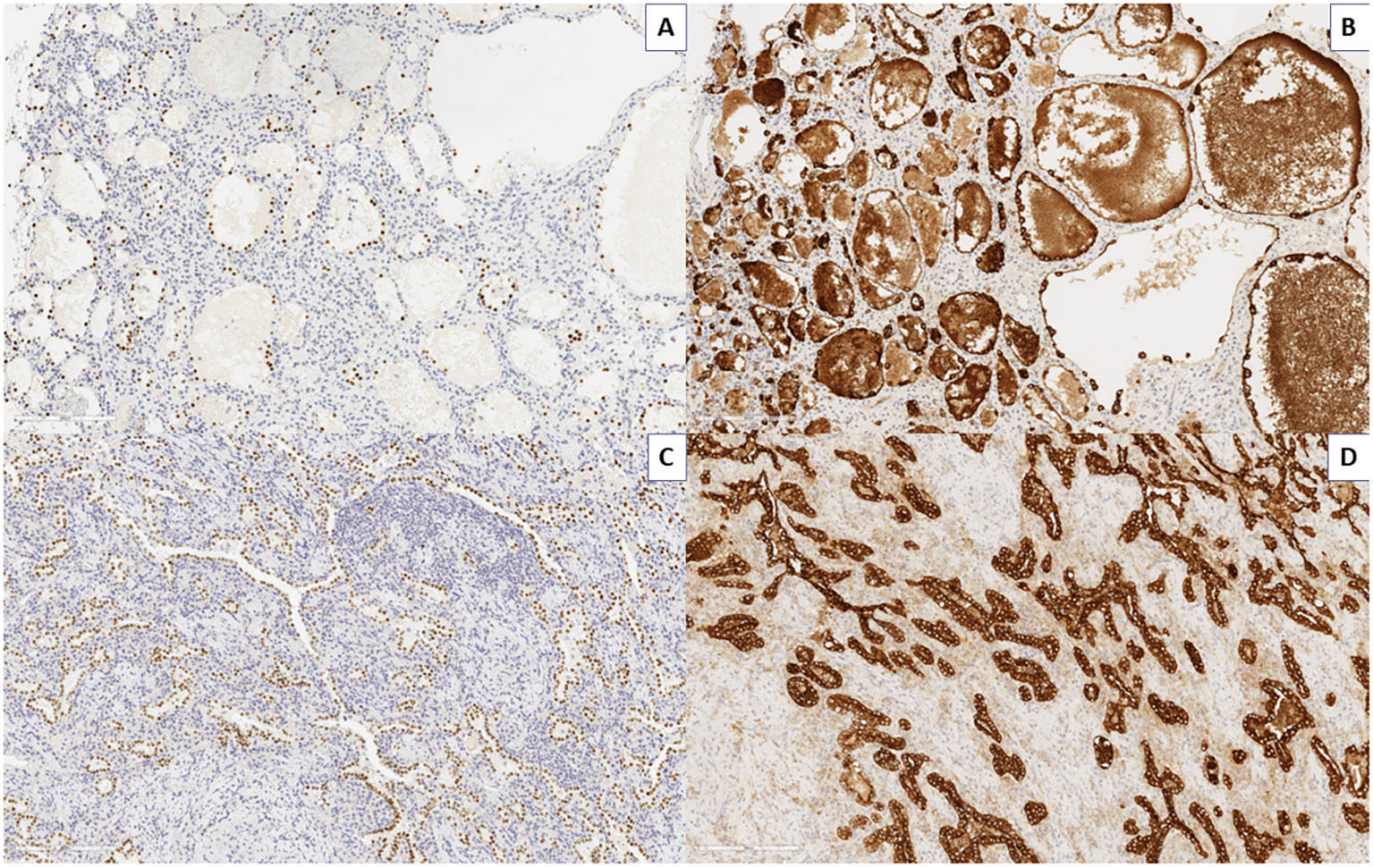
Immunohistochemistry revealed, in both cases, strong and diffuse positivity for TTF1 and EMA of the epithelial cist-lining component [**(A)** case 1, TTF1, scale bar: 200 μm; **(B)** case 1, EMA, scale bar: 200 μm; **(C)** case 2, TTF1, scale bar: 200 μm; **(D)** case 2, EMA, scale bar: 200 μm].

On immunohistochemistry, the stromal spindle cells showed patchy CD34 and vimentin expression only (**Figure 2**), while the cyst-lining cells strongly expressed broad-spectrum cytokeratins (CK MNF116), epithelial membrane antigen (EMA), and thyroid transcription factor-1 (TTF-1), consistent with an alveolar epithelial phenotype. They did not express GATA3 binding protein, CD34, or vimentin (**Figure 3**). Considering the histological features and immunohistochemical profile, a diagnosis of alveolar adenoma was confirmed. The tumor was completely excised. No further follow-up for the lung nodule was needed, albeit the patient remained under clinical surveillance due to the breast carcinoma. No alterations in any of the genes tested were identified by NGS.

Case 2. A 35-year-old female, with a history of heart transplantation due to dilative cardiomyopathy and end-stage chronic kidney disease awaiting kidney transplantation, underwent a PET-CT scan to rule out a suspected lymphoproliferative disorder, prompted by elevated Epstein-Barr Virus-DNA level. Medical history of the patient also included L3-L4 disc herniations with multiple disc protrusions, mild papular facial rosacea with telangiectasias and metrorrhagia treated by uterine curettage. The PET-CT scan evidenced a well-circumscribed nodular thickening of the right lower lobe showing mild hypermetabolism (SUVmax: 3.3). A subsequent chest CT demonstrated an 11 mm hypodense subpleural nodule with slightly blurred margins, connected to a satellite pleural thickening, with no evidence of lymphadenopathy. Further diagnostic workup was undertaken, and a wedge resection of the lesion and a mediastinal lymphadenectomy were performed.

Gross examination evidenced a 0.6 cm lesion. Histologically, the tumor was composed of multiple cystic spaces, filled with eosinophilic material, lined by cuboidal epithelial cells. The surrounding stroma consisted of sporadic, mildly atypical, spindle cells admixed with an inflammatory component. No significant mitotic activity, necrosis, heterologous elements (i.e. cartilage, adipose tissue, smooth muscle), invasive growth or lymph node metastases were present (**Figure 1**).

From an immunohistochemical point of view, epithelial cells showed positivity for the cytokeratin MNF116 and thyroid transcription factor 1 (TTF1) (**Figure 3**), while stromal cells showed focal and weak positivity for vimentin, CD34, smooth muscle actin and S100 (**Figure 2**). No alterations in any of the genes tested were identified by NGS.

## Discussion

At our institution, the incidence of alveolar adenoma was 0.2%, further supporting the rarity of this entity, which was only reported approximately seventy times in literature (**Supplementary Table 1**). However, the true incidence may be even higher, as many of these nodules are incidentally detected due to their asymptomatic or subclinical nature. Molecular investigations have been limited to a few anecdotal reports. Cavazza et al. (2) described microsatellite instability in the epithelial component, whereas an earlier study by Roger et al. reported a diploid DNA pattern with an unbalanced t(10;16) translocation. In both of our cases, NGS did not detect any molecular alteration, including microsatellite analysis. This apparent discrepancy may be attributable to differences in sampling or analyzed compartments, or technical differences in the methodologies employed. Nevertheless, alveolar adenoma remains a benign neoplasm for which surgical resection appears to be curative.

The main challenge, however, lies in the differential diagnosis with other pulmonary lesions that may entail a completely different clinical management, particularly when the lesion demonstrates FDG uptake on PET-CT, as observed in one of our cases. Although PET positivity has only rarely been reported (Burke LM, Hum Pathol1999 (3); Fujimoto K et al., Pathol Int 2008 (4); Nosotti M et al., J Cardiothorac Surg 2012 (5), even low-level uptake may raise concern for malignancy and prompt more aggressive diagnostic and therapeutic approaches.

In this context, the most relevant differential diagnosis is represented by well-differentiated pulmonary adenocarcinoma, particularly the lepidic subtype, which may share overlapping radiological and morphological features. However, in contrast to adenocarcinoma, alveolar adenoma lacks cytologic atypia, mitotic activity, and any evidence of invasive growth. Histologically, it is characterized by a well-circumscribed lesion composed of cystic or microcystic spaces lined by a single layer of cuboidal type II pneumocytes, typically expressing TTF1, and supported by a myxoid or variably fibrous stroma.

Other entities may enter the differential diagnosis. Atypical adenomatous hyperplasia and adenocarcinoma *in situ* can show proliferation of TTF1-positive pneumocytes, but they usually present as smaller, non-cystic lesions with a purely lepidic growth pattern and at least focal cytologic atypia. Sclerosing pneumocytoma may also express TTF1 and derive from primitive respiratory epithelium, yet it is distinguished by its dual cell population and characteristic architectural heterogeneity, including papillary, solid, sclerotic, and hemorrhagic patterns. Pulmonary hamartoma, although often well circumscribed, typically contains a mixture of cartilage, adipose tissue, and fibrous elements, frequently associated with characteristic radiologic findings that are not observed in alveolar adenoma. Bronchiolar adenoma (ciliated muconodular papillary tumor) may also be considered, but the presence of a bilayered epithelium with basal cells and mucinous or ciliated components allows distinction from the simple pneumocytic lining of alveolar adenoma. In addition, cystic developmental lesions such as congenital pulmonary airway malformation, as well as rare metastatic tumors with cystic change, may be considered in selected clinical settings, although their clinicopathological context usually facilitates the distinction.

From an immunohistochemical standpoint, the expression of TTF1 supports pneumocyte differentiation but lacks specificity, being shared with a wide spectrum of primary lung neoplasms. Therefore, the diagnosis ultimately relies on the combination of morphological features, including the absence of atypia and invasion, and the characteristic stromal component, often myxoid and paucicellular.

Importantly, the benign nature of alveolar adenoma, together with its typically indolent behavior and the observation that FDG uptake, when present, tends to be low, suggests that a conservative surgical approach should be considered. In this setting, parenchyma-sparing resections, such as wedge resection or limited segmentectomy, may represent the most appropriate management strategy, allowing definitive diagnosis and treatment while preserving lung function.

Awareness of this entity is therefore crucial to avoid overtreatment, particularly in cases where radiological findings or PET positivity might otherwise lead to more extensive resection.
