# Supplementary material for: Case Report: Pulmonary alveolar adenoma: a case series from a single institution and literature review
Source: Front Oncol. 2026 Jun 29;16:1887451. doi: 10.3389/fonc.2026.1887451 (PMC13357278; doi:10.3389/fonc.2026.1887451)
Supplement: Supplementary file 1 [file Table1.pdf]

Authors	Year	Cases	Age	Sex	Risk factors and comorbidties	Presentation	Radiology	Location	Size	Treatment	F/U (months)
This case series	2026	2	55	F	Non-smoker, obesity, breast carcinoma	Asymptomatic	CT: solitary, peripheral nodule<br>PET: No uptake	LLL	1.8	Segmentectomy and lymphadenectomy	1
			36	F	Post-myocardic dilative cardiomyopathy, end-stage chronic kidney disease	Asymptomatic	CT: Hypodense, subpleural nodule and satellite thickening of the pleura<br>PET: Solitary nodular thickening (SUVmax: 3.3)	RLL	1.1	Wedge resection and lymphadenectomy	11 (death for other reasons)
Lovrenski A [6]	2024	5	52	M	NA	Shoulder pain	CT: Solitary, peripheral nodule	RUL	3.0	Wedge biopsy	18-36
			61	F	NA	Shortness of breath	CT: Solitary, peripheral nodule	RLL	2.2	Wedge biopsy	18-36
			68	F	NA	Dyspnoea	CT: Solitary, peripheral nodule	RLL	1.4	Wedge biopsy	18-36
			66	F	NA	Back pain, dyspnoea	CT: Solitary, peripheral nodule	LLL	2.5	Wedge biopsy	18-36
			53	F	NA	Asymptomatic	CT: Solitary, peripheral nodule	RUL	2.0	Wedge biopsy	18-36
Popovic K [7]	2023	1	69	F	Non smoker, family history of lung cancer, renal clear cell carcinoma, PCOS, liver haemangioma	Asymptomatic	CT: Solitary, well-demarcated nodule	RLL	1.2	Wedge resection	24
Sung YE [8]	2023	1	37	F	NA	Chest discomfort	CT: Solitary, peripheral cyst	LLL	9.0	Wedge biopsy	24
Zhou G [9]	2023	1	46	F	NA	Asymptomatic	CT: Solitary nodule<br>PET: No uptake	LLL	3.0	Lobectomy and lymphadenectomy	NA
Lu YW [10]	2022	1	52	F	NA	Asymptomatic	CT: Solitary, peripheral nodule<br>PET: No uptake	RUL	1.2	Wedge biopsy	36
Garcia - Abellas P [11]	2022	1	48	F	NA	Dust-associated pneumonia	CT: Solitary, cystic, peripheral nodule	RLL	3.0	Segmentectomy	6
Yilmaz A [12]	2022	1	51	F	Non-smoker	Chest pain	X-ray: Solitary nodule<br>CT: Solitary, well-circumscribed, nodule	RUL	1.8	Wedge resection	NA
Roshkovan L [13]	2020	1	48	F	Non-smoker, colonic tubulovilloud adenoma, pituitary adenoma	Chest pain	CT: smoothly marginated, solid-appearing, juxta-pleural nodule<br>PET: SUVmax 0,6	LUL	1.2	Wedge resection	NA

Volk L [14]	2020	1	26	F	NA	Left sided pneumothorax	X-ray: Left-sided penumothorax, blebs, mediastinal shift to the right<br>CT: Extensive bullous disease of the left upper lobe	LUL	na	Lobectomy	NA
Kavas M [15]	2019	4	36	M	NA	Chest pain	CT: Nodule without calcification<br>PET: SUVmax 2	LLL	2.6	Thoracotomy	34
			51	F	NA	Shortness of breath	X-ray: increased density of 2 cm<br>CT: Solitary nodule	RUL	1.8	Wedge resection	180
			38	F	NA	Thoracic hemoptysis and pain	CT: Solitary nodule	RUL	1.3	Wedge resection	120
			59	F	NA	Left shoulder pain	CT: Solitary nodule	LLL	2.0	Wedge resection	96
Gan M [16]	2019	1	48	F	Mild cerebral infarction	Asymptomatic	CT: Solitary mass	LLL	3.5	Lobectomy	60
Zhang X [17]	2017	1	40	M	Pharyngitis	Cough and expectoration	CT: Mass with clear border and uniform density	LLL	5.1	Lobectomy	26
Okada S [18]	2016	1	83	M	Ex-smoker (40 pach-years)	NA	X-ray: Solitary nodule<br>CT: Well-circumscribed, calcified nodule<br>PET: SUVmax 1.4	LUL	1.8	Segmentectomy	48
Hsieh M [19]	2015	1	67	M	Diabetes, hypertension	Dysphagia and nausea	CT: single, large, unicystic lesion	RLL	3.9	Wedge resection	NA
Tang X [20]	2015	1	47	F	Non-smoker, uterine myoma, intracranial vascular malfomartion	Asymptomatic	CT: Solitary, peripheral, cystic nodule	RLL	4.0	Segmentectomy	52
Yamamoto Y [21]	2015	1	65	F	Breast cancer	Asymptomatic	CT: Double bassel-shaped nodule with meniscus air density	LLL	1.3	Segmentectomy	NA
Lee DS [22]	2014	1	57	F	Thyroid nodule	Cough	X-ray: LLL nodule<br>CT: RUL nodule	LLL,RUL	1.6-0.8	Wedge resection	NA
Kazerouni H [23]	2013	1	41	F	NA	Asymptomatic	X-ray: Well-circumscribed lesion<br>CT: enhancing mass<br>PET: Negative	LLL	1.3	Lobectomy	NA
Wang L [24]	2013	1	48	F	Non-smoker	Asymptomatic	CT: solitary peripheral mass	RLL	4.0	Lobectomy	48

Wang X [25]	2013	1	60	F	Non-smoker, hypertension, diabetes, renal calculus	Asymptomatic	CT: Solitary nodule<br>MRI: T1 hypointensity, T2 hyperintensity	RLL	7.3	Wedge biopsy	6
De Rosa [26]	2012	2	24	M	Non-smoker	NA	X-ray: Solitary peripheral nodule<br>CT: Well-circumscribed, homogeneous non-calcified mass with contrast enhancement in intranodular areas<br>PET: Negative	LLL	NA	Wedge resection	7
			35	F	Non-smoker	Right sided pleuritic chest pain	X-ray: Single nodule<br>CT: Well-circumscribed, pleural-based nodule	RUL	5.0	Wedge resection	132
Nosotti M [5]	2012	1	54	F	NA	Dry cough, dyspnoea	CT: Solitary central nodule<br>PET: SUVmax 1.06	LLL	1.8	Lobectomy	12
Panagiotou I [27]	2012	1	42	F	Ex-smoker (40 pack-years), familiar history of lung cancer	Asymptomatic	CT: Solitary peripheral nodule	RLL	1.5	Wedge biopsy	12
Bahvsar T [28]	2011	1	59	F	Smoker, COPD, poorly differentiated carcinoma	Asymptomatic	CT: Single mass<br>PET: FDG avidity	RUL	0.2	Lobectomy	NA
Kondo N [29]	2011	1	61	F	NA	Asymptomatic	CT: Solitary peripheral nodule<br>PET: No uptake	LUL	2.4	Segmentectomy	12
Petrella F [30]	2010	1	38	F	NA	Dyspnoea	CT: Solitary, multiseptated cystic mass	LLL	9.1	Wedge biopsy	NA
Glaab R [31]	2009	1	58	M	Ex-smoker	Hemoptysis	X-ray: Well-defined nodule<br>CT: Solid-heterogeneus, non-calcified, well-defined nodule	RLL	4.2	Segemntectomy	NA
Nakamura H [32]	2009	1	58	F	NA	Asymptomatic	CT: Solitary nodule	LUL	0.8	Segmentectomy	3
Gonzalez E [33]	2008	1	71	M	Prostatic adenocarcinoma	Asymptomatic	CT: Solitary, well-circumscribed, peripherally located nodule	RLL	1.5	Segmentectomy	NA
Sak SD [34]	2007	2	62	M	NA	Chest pain	CT: Solitary peripheral mass	LLL	1.5	Wedge biopsy	22
			54	M	NA	Asymptomatic	X-ray: Solitary mass	LLL	4.0	Wedge biopsy	32
Saito EH [35]	2006	1	35	F	Non-smoker	Dry cough, dyspnoea	CT: Solitary, peripheral cavitation nodule	RUL	2.0	Wedge biopsy	NA

Halldorsson A [36]	2005	1	43	M	NA	Pleuritic chest pain	CT: Solitary peripheral nodule<br>PET: No uptake	LLL	1.1	Wedge biopsy	18
Cavazza A [2]	2004	1	69	F	NA	Asymptomatic	CT: Solitary peripheral cystic nodule	RUL	3.5	Wedge biopsy	13
Golubovic M [37]	2004	1	64	F	Diabetes, heart diseases (not specified)	Asymptomatic	X-ray: Well-marked solitary change<br>CT: Solitary well-marked shadow	LUL	2.27 - 4	Surgery	96
Hartman MS [38]	2004	1	51	F	NA	Dry cough	X-ray: Solitary nodule	RUL	3.4	Segmentectomy	18
Cakan [39]	2003	1	34	F	Non-smoker	Chest pain	X-ray: Coin lesion<br>CT: Solitary pulmonary nodule	LUL	1.8	Wedge resection	NA
Papla B [40]	2003	2	54	M	NA	Asymptomatic	X-ray: Solitary nodule	RL	2.5	Wedge biopsy	144
			66	F	Metallic dust exposure	Asymptomatic	X-ray: Solitary nodule	RML	1.4	Surgery	NA
Fujimoto K [4]	2002	1	47	F	NA	Productive cough	X-ray: Three nodular opacities<br>CT: Three nodules with well-defined, smooth margins<br>MRI: low-signal intensity with a peripheral rim of moderate T1-intensity and low signal T2-intensity	LLL	NA	Wedge resection	15
Burke L [3]	1999	10	45	M	NA	NA	NA	LUL	1.5	NA	NA
			58	M	NA	Asymptomatic	NA	LLL	1.9	NA	NA
			50	M	NA	Asymptomatic	NA	LLL	NA	NA	NA
			39	M	NA	Asymptomatic	NA	TLLL	2.0	NA	NA
			41	F	Melanoma	Asymptomatic	NA	LLL	1.1	NA	NA
			52	F	NA	Asymptomatic	NA	LLL	3.0	NA	NA

			41	F	NA	Asymptomatic	NA	LLL	2.5	NA	NA
			45	F	NA	Asymptomatic	NA	LLL	2.0	NA	NA
			46	F	Uterine leiomyoma	Asymptomatic	NA	NA	NA	NA	NA
			NA	NA	NA	Lack of appetite	NA	RL	3.0	NA	NA
Böhm J [41]	1997	1	52	F	Cervical spine syndrome	Asymptomatic	X-ray: Solitary peripheral nodule	LLL	2.0	Wedge resection	12
Oliveira P, Roque L [42,43]	1996	1	55	F	NA	Chronic non-productive cough	X-ray: Wll-circumscribed mass<br>CT: Well-circumscribed mass	RLL	6.0	Segmentectomy	32
Semeraro D [44]	1989	1	67	F	Non-smoker, chest infection	NA	X-ray: Circumscribed lesion	RML	2.8	Thoracotomy	NA
Al-Hilli F [45]	1987	1	60	F	Mild aortic stenosis	Dyspnoea, left-sided chest pain	X-ray: Solitary uniform non-calcified nodule<br>CT: Solitary uniform non-calcified nodule	Lingula	1.0	Thoracotomy	NA
Yousem SA [1]	1986	6	45	F	NA	Asymptomatic	X-ray: Solitary nodule	LLL	2.0	Wedge biopsy	13
			54	F	NA	Asymptomatic	X-ray: Solitary nodule	RUL	2.5	Lobectomy	12
			58	M	NA	Asymptomatic	X-ray: Solitary nodule	LLL	1.5	Wedge biopsy	56
			59	F	NA	Asymptomatic	X-ray: Solitary nodule	RUL	1.3	Lobectomy	13
			64	M	NA	Asymptomatic	X-ray: Solitary nodule	RUL	1.2	Lobectomy	NA
			74	F	NA	Weakness, rash	X-ray: Solitary nodule	RML	2.5	Lobectomy	120

Supplementary table 1. Clinical features of alveolar adenomas reported in literature. Abbreviations: F/U, follow-up; NA, not available; CT, computer tomography, LLL, left lower lobe; LUL, left upper lobe; RUL, right upper lobe; RML, right middle lobe; RLL, right lower lobe.

Bibliography

[1] Yousem SA, Hochholzer L. Alveolar adenoma. Hum Pathol 1986;17:1066–71. https://doi.org/10.1016/S0046-8177(86)80092-2.

[2] Cavazza A, Paci M, De Marco L, Leporati G, Sartori G, Bigiani N, et al. Alveolar Adenoma of the Lung: A Clinicopathologic, Immunohistochemical, and Molecular Study of an Unusual Case. Int J Surg Pathol 2004;12:155–9. https://doi.org/10.1177/106689690401200212.

[3] Burke LM, Rush WI, Khoor A, Mackay B, Oliveira P, Whitsett JA. Alveolar adenoma: a histochemical, immunohistochemical, and ultrastructural analysis of 17 cases. Hum Pathol 1999;30:158–67. https://doi.org/10.1016/s0046-8177(99)90270-8.

[4] Fujimoto K, Müller NL, Sadohara J, Harada H, Hayashi A, Hayabuchi N., et al. Alveolar adenoma of the lung: computed tomography and magnetic resonance imaging findings. J Thorac Imaging 2002;17:163–6. https://doi.org/10.1097/00005382-200204000-00011.

[5] Nosotti M, Mendogni P, Rosso L, Tosi D, Palleschi A, Basciu M, et al. Alveolar adenoma of the lung: unusual diagnosis of a lesion positive on PET scan. A case report. J Cardiothorac Surg 2012;7:1. https://doi.org/10.1186/1749-8090-7-1.

[6] Lovrenski A, Gardić N, Šunjević M, Dragišić D, Vučković D. Alveolar Adenoma: A 20-Year Experience at a Western Balkan University Hospital and a Literature Review. Int J Surg Pathol 2024;32:982–91. https://doi.org/10.1177/10668969231204998.

[7] Popovic K, Miladinović M, Vučković L, Nedović Vuković M. Rare benign lung tumours presenting with high clinical suspicion for malignancy: a case series and review of the literature. Folia Histochem Cytobiol 2023;61:130–42. https://doi.org/10.5603/FHC.a2023.0011.

[8] Sung YE, Moon MH. Enlarging pulmonary cyst: a rare form of alveolar adenoma. J Cardiothorac Surg 2023;18:303. https://doi.org/10.1186/s13019-023-02409-9.

[9] Zhou G, Estrella Perez Y, Leng B. Alveolar Adenoma With CD34 Diffusely Positive Stromal Cells: A Rare Case Report and Literature Review. J Investig Med High Impact Case Rep 2023;11:23247096231181959. https://doi.org/10.1177/23247096231181959.

[10] Lu Y-W, Chang S-L, Yeh Y-C, Hsieh Y. Alveolar adenoma and coexisting atypical adenomatous hyperplasia: a case report and literature review. Pathologica 2022;114:326–31. https://doi.org/10.32074/1591-951X-755.

[11] García-Abellás P, Alarcón-Rodríguez J. Alveolar adenoma: Atypical radiological presentation of a rare neoplasm. Radiol Engl Ed 2022;64:589–90. https://doi.org/10.1016/j.rxeng.2022.10.004.

[12] Yilmaz A, Bayramgürler B, Aksoy F, Ünver E, Düzgün S. 78-80 Alveolar Adenoma n.d.

[13] Roshkovan L, Thompson JC, Katz SI, Deshpande S, Jenkins T, Nowak AK, et al. Alveolar adenoma of the lung: multidisciplinary case discussion and review of the literature. J Thorac Dis 2020;12:6847–53. https://doi.org/10.21037/jtd-20-1831.

[14] Volk L, Minerowicz C, Saadat S, Langenfeld JE. Multicystic Alveolar Adenoma in a Symptomatic Adult with Extensive Bullae and Mediastinal Shift. Surg Case Rep 2020;2020:1–3. https://doi.org/10.31487/j.SCR.2020.03.03.

[15] Kavas M, Öztürk A, Derdiyok O, Atinkaya C, Ürek Ş, Yılmaz A et al. Rare Lung Tumors: Alveolar Adenoma-Four Case Reports. Turk Thorac J 2019;20:203–5. https://doi.org/10.5152/TurkThoracJ.2018.18015.

[16] Gan M, Weng S, Zheng H, Zhang L. Coexistence of lung alveolar adenoma with bronchogenic cyst: a case report and literature review n.d.

[17] Zhang X, Bai Y, Wang X, Huang K. Alveolar adenoma with the round-shaped mesenchymal cells: a rare case and review of literature n.d.

[18] Okada S, Ohbayashi C, Nishimura M, Abe K, Choh S, Shimada J, et al. Malignant transformation of alveolar adenoma to papillary adenocarcinoma: a case report. J Thorac Dis 2016;8:E358–61. https://doi.org/10.21037/jtd.2016.03.37.

[19] Hsieh M-S, Tseng Y-H, Hua S-F, Chou YH. Cystic alveolar adenoma: an unusual clinical presentation of a rare lung neoplasm. Pathology (Phila) 2015;47:78–80. https://doi.org/10.1097/PAT.0000000000000201.

[20] Tang X, Wu Z, Shen Y. Coexistence of lung alveolar adenoma with cerebral arteriovenous malformations: A case report and literature review. Oncol Lett 2015;10:250–4. https://doi.org/10.3892/ol.2015.3225.

[21] Yamamoto M, Mukaida H, Takiyama W, Egawa H, Kaneko M. A Case of Alveolar Adenoma of the Lung. Nihon Rinsho Geka Gakkai Zasshi J Jpn Surg Assoc 2016;77:312–6. https://doi.org/10.3919/jjsa.77.312.

[22] Lee DS, Hwang M, Lim J, Kim M, Jung B, Kang G, et al. A Case of Alveolar Adenoma Involving Multiple Lung Nodules. Korean J Med 2014;86:623. https://doi.org/10.3904/kjm.2014.86.5.623.

[23] Hamid Kazerouni A, Chetty R. Alveolar adenoma of the lung. Diagn Histopathol 2013;19:311–3. https://doi.org/10.1016/j.mpdhp.2013.06.012.

[24] Wang L, Wang X, Rustam A, et al. Alveolar Adenoma Resected by Thoracoscopic Surgery. Ann Thorac Cardiovasc Surg 2013;19:489–91. https://doi.org/10.5761/atcs.cr.13-00118.

[25] Wang X, Li W-Q, Yan H-Z, Hu J. Alveolar adenoma combined with multifocal cysts: Case report and literature review. J Int Med Res 2013;41:895–906. https://doi.org/10.1177/0300060513477304.

[26] De Rosa N, Maiorino A, De Rosa I, Curcio C, Sellitto C, Amore D. CD34 Expression in the Stromal Cells of Alveolar Adenoma. Case Rep Med 2012;2012:913517. https://doi.org/10.1155/2012/913517.

[27] Panagiotou I, Kostikas K, Sampaziotis D, Kotoulas C. Alveolar adenoma: an extremely rare innocent coin lesion. Interact Cardiovasc Thorac Surg 2012;14:335–7. https://doi.org/10.1093/icvts/ivr040.

[28] Bhavsar T, Uppal G, Travaline JM, Gaughan C, Huang Y, Khurana J. An unusual case of a microscopic alveolar adenoma coexisting with lung carcinoma: a case report and review of the literature. J Med Case Reports 2011;5:187. https://doi.org/10.1186/1752-1947-5-187.

[29] Kondo N, Torii I, Hashimoto M, Takuwa T, Tanaka F, Tsujimura T, et al. Alveolar Adenoma of the Lung: A Case Report. Ann Thorac Cardiovasc Surg 2011;17:71–3. https://doi.org/10.5761/atcs.cr.09.01504.

[30] Petrella F, Rizzo S, Pelosi G, Borri A, Galetta D, Gasparri R, et al. Giant Alveolar Adenoma Causing Severe Dyspnoea. J Thorac Oncol 2010;5:1088–90. https://doi.org/10.1097/JTO.0b013e3181d95ca5.

[31] Glaab R, Turina M, Achermann E, Maurer R, Went P, Schöb O. Alveolar adenoma--a rare pulmonary mass: case report and review of the literature. Zentralbl Chir 2009;134:478–80. https://doi.org/10.1055/s-0028-1098763.

[32] Nakamura H, Adachi Y, Arai T, Miwa K, Haruki T, Fujioka S, et al. A Small Alveolar Adenoma Resected by Thoracoscopic Surgery. Ann Thorac Surg 2009;87:956–7. https://doi.org/10.1016/j.athoracsur.2008.07.078.

[33] González ET, Sánchez-Yuste R, Jiménez-Heffernan JA. Cytologic features of pulmonary alveolar adenoma. Acta Cytol 2008;52:739–40. https://doi.org/10.1159/000325634.

[34] Sak SD, Koseoglu RD, Demirag F, Akbulut H, Gungor A. Alveolar adenoma of the lung: Immunohistochemical and flow cytometric characteristics of two new cases and a review of the literature. APMIS 2007;115:1443–9. https://doi.org/10.1111/j.1600-0463.2007.00762.x.

[35] Saito EH, de Aaraujo LR, Carneiro LH, De Oliveira Neto A, Correa JC, Teixeira LSC. Alveolar adenoma. J Bras Pneumol Publicacao Of Soc Bras Pneumol E Tisilogia 2006;32:267–9. https://doi.org/10.1590/s1806-37132006000300014.

[36] Halldorsson A, Dissanaike S, Kaye KS. Alveolar adenoma of the lung: a clinicopathological description of a case of this very unusual tumour. J Clin Pathol 2005;58:1211–4. https://doi.org/10.1136/jcp.2004.020800.

[37] Golubović M, Vučković L, Klem I, Eri Z, Savjak D. Alveolar adenoma of lung: A case report. Arch Oncol 2004;12(Suppl 1):69.

[38] Hartman MS, Epstein DM, Geyer SJ, Keenan R. Alveolar Adenoma. Ann Thorac Surg 2004;78:1842–3. https://doi.org/10.1016/j.athoracsur.2003.07.015.

[39] Cakan A, Samancilar O, Nart D, Cagiric U. Alveolar adenoma: an unusual lung tumor. Interact Cardiovasc Thorac Surg 2003;2:345–7. https://doi.org/10.1016/S1569-9293(03)00067-7.

[40] Papla B, Malinowski E. Alveolar adenoma of the lung--a report of two cases. n.d.

[41] Böhm J, Fellbaum C, Bautz W, Präuer HW, Höfler H . Pulmonary nodule caused by an alveolar adenoma of the lung. Virchows Arch 1997;430:181–4. https://doi.org/10.1007/BF01008040.

[42] Oliveira P, Almeida MO, Moura Nunes JF, Clode AL, Duro Da Costa J. Alveolar adenoma of the lung: further characterization of this uncommon tumour. Virchows Arch 1996;429–429. https://doi.org/10.1007/BF00192432.

[43] Roque L, Oliveira P, Martins C, Carvalho C, Serpa A, Soares J. A nonbalanced translocation (10;16) demonstrated by FISH analysis in a case of alveolar adenoma of the lung. Cancer Genet Cytogenet 1996;89:34–7. https://doi.org/10.1016/0165-4608(95)00309-6.

[44] Semeraro D, Gibbs AR. Pulmonary adenoma: a variant of sclerosing haemangioma of lung? J Clin Pathol 1989;42:1222–3. https://doi.org/10.1136/jcp.42.11.1222.

[45] Al-Hilli F. Lymphangioma (or alveolar adenoma?) of the lung. Histopathology 1987;11:979–80. https://doi.org/10.1111/j.1365-2559.1987.tb01904.x.
